# Placental Microparticles and MicroRNAs in Pregnant Women with *Plasmodium falciparum* or HIV Infection

**DOI:** 10.1371/journal.pone.0146361

**Published:** 2016-01-12

**Authors:** Laura Moro, Azucena Bardají, Eusebio Macete, Diana Barrios, Diana M. Morales-Prieto, Carolina España, Inacio Mandomando, Betuel Sigaúque, Carlota Dobaño, Udo R. Markert, Daniel Benitez-Ribas, Pedro L. Alonso, Clara Menéndez, Alfredo Mayor

**Affiliations:** 1 ISGlobal, Barcelona Centre for International Health Research (CRESIB), Hospital Clínic—Universitat de Barcelona, Barcelona, Spain; 2 Centro de Investigação em Saúde da Manhiça, Manhiça, Mozambique; 3 Placenta-Labor, Department of Obstetrics, University Hospital Jena, Jena, Germany; 4 Department of Gastroenterology, Centro de Investigación Biomédica en Red de Enfermedades Hepáticas y Digestivas (CIBERehd), Hospital Clínic, Barcelona, Spain; Institut de Recherche pour le Développement, FRANCE

## Abstract

**Background:**

During pregnancy, syncytiotrophoblast vesicles contribute to maternal tolerance towards the fetus, but also to pathologies such as pre-eclampsia. The aim of the study was to address whether *Plasmodium falciparum* and HIV infections in pregnancy affect the secretion, microRNA content and function of trophoblast microparticles.

**Methods:**

Microparticles were isolated and characterized from 122 peripheral plasmas of Mozambican pregnant women, malaria- and/or HIV-infected and non-infected. Expression of placenta-related microRNAs in microparticles was analysed by qPCR and the effect of circulating microparticles on dendritic cells assessed by phenotype analysis and cytokine/chemokine measurement.

**Results:**

Concentrations of total and trophoblast microparticles detected by flow cytometry were higher in HIV-positive (P = 0.005 and P = 0.030, respectively) compared to non-infected mothers, as well as in women delivering low birthweight newborns (P = 0.032 and P = 0.021, respectively). miR-517c was overexpressed in mothers with placental malaria (P = 0.034), compared to non-infected. Microparticles from HIV-positive induced a higher expression of MHCII (P = 0.021) and lower production of MCP1 (P = 0.008) than microparticles from non-infected women.

**Conclusions:**

In summary, alterations in total and trophoblast microparticles associated with malaria and HIV in pregnant women may have an immunopathogenic role. The potential for placental-derived vesicles and microRNAs as biomarkers of adverse outcomes during pregnancy and malaria infection should be confirmed in future studies.

## Introduction

Infectious pathogens can alter the release and function of extracellular vesicles (EVs) to promote growth and induce transmission, evade host immune system and manipulate cellular microenvironment [1,2]. Exosomes, 30–100 nm and originated from inward budding of endosomal membranes, and microparticles (MPs), 100–1000 nm and originated from outward budding of plasma membrane [3], participate in intercellular communication, including immune functions, in physiological and pathological conditions [1,4]. Key players in functions mediated by EVs are secreted microRNAs (miRNAs), small non-coding RNAs that trigger either mRNA degradation or translational repression [5]. These EVs and miRNAs can be measured in body fluids and present a potential as diagnostic biomarkers [6,7], although standardized procedures are lacking [8].

Little is known about the role of EVs in malaria and human immunodeficiency virus (HIV) infections, two of the major health priorities especially for pregnant women and their infants. Circulating plasma and red blood cell-derived MPs levels increase with malaria severity [9], especially in cerebral malaria [10], and lead to the activation of brain endothelium [11,12]. Genetically deficient mice with impaired capacity to vesiculate are completely resistant to cerebral malaria [13], thus confirming the implication of EVs in the physiopathology of the disease. Importantly, exosome-like vesicles seem to be implicated in cell-to-cell communication between *Plasmodium*-infected red blood cells for the initiation of parasite sexual differentiation [14], and exosomes from *Plasmodium yoelii*-infected reticulocytes elicit protective immune responses against lethal infection in mice [15]. EVs can also contribute to viral infection and pathogenesis [16], such as in the case of HIV, by facilitating macrophage infection [17], inhibiting antigen-presenting cells responsible for early pathogen detection (i.e., dendritic cells (DCs) [18]), and transferring the HIV co-receptor chemokine receptor 5 (CCR5) to other cells [19].

EVs also have an important role in the communication between fetus and maternal immune cells necessary for pregnancy success [20]. Syncytiotrophoblast-derived MPs contribute to maternal tolerance towards the fetus [21,22], but can lead to pre-eclampsia-associated inflammation [23]. Trophoblastic MPs (trMPs) contain placental miRNAs, also present in maternal plasma [24] and breast milk [25], trophoblast cells from placenta or cultured lines [26], and exosomes from villous trophoblasts [27]. Placenta-derived miRNAs [24,27] participate in fine-tuning of gene expression in the fetal-maternal interface [28] and are altered in pregnancy-related pathologies such as pre-eclampsia, intrauterine growth restriction (IUGR) or preterm delivery [26]. However, no previous study has investigated the relationship of trophoblast-derived vesicles with malaria and HIV infections in pregnant women. We hypothesized that modifications in the placenta caused by *Plasmodium falciparum* [29] and HIV [30,31] affect trMP secretion contributing to altered pregnancy immune responses. Therefore, our primary objective was to study *ex-vivo* the effect of *P*. *falciparum* and HIV infections on trMPs levels and miRNA content in Mozambican pregnant women and, secondary, to assess their immunoregulatory role on DCs *in vitro*.

## Materials and Methods

### Study population

This study was nested in a placebo-controlled trial of intermittent preventive treatment in pregnancy (IPTp) with sulphadoxine-pyrimethamine (SP) for malaria prevention conducted at the Manhiça Health Research Centre, Mozambique, between 2003–2006 (NCT00209781) [32]. Malaria transmission is perennial with some seasonality and *P*. *falciparum* is the predominant species [32]. Women were enrolled at the antenatal clinics of the Manhiça District Hospital if they had gestational age ≤28 weeks, not reported allergies to sulpha drugs and were permanent residents of the study area. After providing written informed consent, women were randomized to receive SP or placebo and screened for HIV if they agreed [32]. The study was approved by the Mozambican National Bioethics Committee and the Hospital Clínic of Barcelona Ethics Review Committee.

Maternal HIV-1 infection was diagnosed with Determine HIV-1/2 (Abbott) and confirmed with Unigold rapid test (Trinity Biotech). At delivery, maternal peripheral blood was collected by venipuncture into ethylenediaminetetraacetic acid vacutainers and thin and thick smears prepared, Giemsa-stained and examined for malarial parasites according to quality-control procedures [33]. Blood was centrifuged and plasma stored at -80°C. Placental biopsy specimens were processed for histological examination and classified following published criteria [29]. Newborn's birthweight, gestational age (by Dubowitz method) and maternal hematocrit (in microcapillary tube) were determined.

The current analysis was conducted in 61 randomly-selected HIV-infected and 61 non-infected mothers at delivery receiving either placebo or SP from whom demographic and clinical data, as well as maternal plasma samples, were available.

### Isolation of vesicles from peripheral plasma

Plasma samples were thawed at 37°C in water bath and platelets and/or cell debris eliminated by centrifugation (10 min at 1000g, room temperature). Isolation of vesicles from plasma by centrifugation at 10.000g [4,16,34] and ultracentrifugation at 100.000g [21,23] for 30 minutes at 4°C were compared in 8 pregnant mothers from the study and one non-pregnant control (woman from non-endemic area). Centrifugation was chosen as the best approach for the separation. Pelleted vesicles were resuspended in double filtered Dulbecco's Phosphate Buffered Saline without Ca^2+^ and Mg^2+^ (DPBS, Lonza). The number of circulating vesicles per μl of plasma was assessed using Perfect-Count Microspheres (Cytognos), a microbead-based single-platform system for absolute counts that includes an internal quality control, according to manufacturer’s protocol.

### Quantification of total and trophoblast MPs

Circulating particles were double stained with Annexin V, that binds to all classes of MPs regardless of cell type [35], and a mouse monoclonal IgG1 against human Pregnancy-Specific Glycoprotein1 (PSG1, trophoblast marker) [35] for analysis and quantification by flow cytometry. Briefly, 250000 MPs (as assessed with Perfect-Count Microspheres) resuspended in 100 μl of DPBS were incubated with 7 μg/ml PSG1-PE (catalog No. sc-59348, Santa Cruz Biotechnology) for 6 minutes, washed with DPBS, resuspended in 100 μl of Annexin buffer and subsequently stained for 15 minutes with 5 μl Annexin V-FITC (catalog No. 556419 BD Biosciences; and catalog No. ANXVF-200T, Immunostep) for immediate analysis. trMPs were identified in a BD LSRFortessa SORP as events with forward scatter patterns between those obtained with fluorescent calibration microspheres of diameters 0.2 and 1 μm (Invitrogen Molecular Probes) that were positive both for Annexin V and PSG1 staining [36], and the number of MPs per μl of sample was calculated. Similarly, all the events in this gate positively stained for Annexin V were considered total circulating MPs. Gate limits were set between 0.2 μm to avoid selecting exosomes (up to 0.1 μm [4]) and 1 μm to avoid selecting apoptotic bodies (>1 μm [37]). The gating strategy is shown in S1 Fig.

### Electron microscopy

Negative staining with uranyl acetate was used for electron microscopy imaging. Briefly, 5 μl of isolated MP sample were loaded in a previously charged metal grid, deposited for 30 min, fixed with 1% glutaraldehyde for 5 minutes, washed with water, stained with 2% uranyl acetate for 1 minute and imaged with JEOL 1010 TEM (JEOL).

### Analysis of placental microRNA content by qPCR

Plasmas from 10 mothers negative both for malaria and HIV, 10 placental malaria-infected and 10 HIV-infected women with similar characteristics to the study population (described in S1 Table) and that presented the highest concentrations of trMPs were selected. No double infections were included. Additionally, plasmas from 5 men from non-endemic area, not infected by malaria neither HIV, were included as negative controls. Potential hemolysis in plasma samples (1:10 diluted), shown to greatly affect miRNA expression [38], was addressed by spectrophotometric analysis using the Harboe’s method [39] with Allen’s correction to calculate hemoglobin concentration. Absence of hemolysis was defined by an absorbance of less than 0.2 at 414 nm [38].

Four human placental miRNAs were selected because of their specific profile in the placenta or maternal plasma, their importance in regulatory processes during the gestation and their participation in several pregnancy disorders: hsa-miR-21, highly expressed at the end of pregnancy; hsa-miR-493, representative of chromosome 14 miRNA cluster (C14MC) whose expression decreases at the end of pregnancy; hsa-miR-517c and hsa-miR-519d, both from the cluster C19MC and highly expressed at the end of pregnancy [26,40,41]. In addition, hsa-miR-191 was included as a plasma normalization control [42].

Circulating MPs were isolated from 200 μl of plasma as previously described. RNA was extracted with miRCURY™ RNA Isolation Kit–Biofluids (Exiqon) following manufacturer’s protocol. Reverse transcription was performed with miRNA specific stem-loop RT primers and TaqMan MicroRNA Reverse Transcription Kit (Applied Biosystems) using 10 ng of total RNA measured by Nanodrop (Thermo Scientific) and following the protocol provided by the supplier. qPCR with specific TaqMan Assays (hsa-miR-21, assay ID 000397; hsa-miR-493-5p, assay ID 001040; hsa-miR-517c, assay ID 001153; hsa-miR-519-3p, assay ID 002403; hsa-miR-191-5p, assay ID 002299) and TaqMan Universal PCR Master Mix (all from Applied Biosystems) was performed using 1.5 μl of reverse transcription product per reaction. All reactions were run in duplicates including no-template controls in 96-well plates on a Mx3005P QPCR System (Applied Biosystems) and threshold cycles (Cts) were obtained after 40 cycles. If miRNA expression was not detected in a sample, a Ct value of 41 was arbitrary assigned. ΔΔCts were calculated using values from non-infected women as calibrators and miR-191 as endogenous control [42]. Total *P*. *falciparum* RNA was included as a negative control.

### *In vitro* stimulation of dendritic cells with MPs

Maternal antigen-presenting cells, including DCs, are important players in this fetal-maternal immune adjustment between stimulatory and tolerogenic responses and are involved in cell-cell interactions in the decidualized endometrium [43]. In addition, DCs have been used in previous MP studies as a model to assess the effect of EVs on immune cell [44,45]. Human DCs were generated from monocytes obtained from buffy coats of healthy donors that provided written informed consent, and grown in serum added-X-VIVO-15 culture medium (Sigma and Lonza, respectively) with interleukin 4 (IL4) and granulocyte macrophage colony-stimulating factor (GM-CSF) (both from Miltenyi Biotec). To evaluate the immunoregulatory properties of MPs, 100.000 DCs in 100 μl of culture medium were incubated for 24h with total MPs obtained from 20 μl plasma of individual women: 8 negative both for malaria and HIV (MP_NI_), 10 HIV-infected (MP_HIV_) and 8 women with placental malaria by histology (MP_malaria_) with similar characteristics to the study population and that presented the highest concentrations of trMPs in plasma. No double infections were included. DCs were incubated in every experiment with 400 ng of lipopolysaccharide (LPS) (Invivogen) as a positive and normalization control, and with cell culture medium alone as a negative control. To determine whether MPs have a synergistic effect on LPS mediated DC-stimulation, a second incubation condition with the same amount of MPs plus 10 ng of LPS was included for each sample.

DC phenotype after *in vitro* stimulation with MPs was analysed using the co-stimulatory and activation markers CD80, CD86 and major histocompatibility complex II (MHCII). Cells were incubated with 1μg/ml of anti-CD80, anti-CD86 and anti-MHCII (BD Biosciences) during 30 minutes at 4°C, washed and stained with secondary polyclonal goat anti-mouse-PE antibody (catalog No. 550589, BD Biosciences) for 30 minutes at 4°C. Fluorescence measurements were performed by flow cytometry in a BD FACSCantoII. Normalization of percentages and mean fluorescence intensities (MFIs) of activation markers with respect to the positive control was performed by arbitrary assigning the value 100% to percentages and 1 to MFIs for the LPS condition in each experiment in order to control for potential technical and donor variabilities.

Cytokine and chemokine concentrations were analysed in 50 μl of post-stimulus culture supernatant using the Cytokine Human 30-plex Panel (Life Technologies) as indicated by the manufacturer. Plates were read with a Luminex xMAPTM (Luminex) and concentrations calculated with respect to the kit standards. Concentrations over or below the limit of quantification of the standard curves were assigned twice the value of the most concentrated standard or half the value of the most diluted one, respectively. The available volume of culture supernatant did not allow including several dilutions, and therefore undiluted supernatants were used for the assay.

### Definitions

Pregnant women were classified as primigravidae (first pregnancy), secundigravidae (second pregnancy) and multigravidae (≥2 previous pregnancies). Age was categorized as ≤20, 20–24 or ≥25 years on the basis of maternal age terciles in this population. Placental and peripheral malaria infections were defined by the presence of parasites by histology in placental sections (active placental infection) [29] or by microscopy in peripheral blood, respectively. Maternal anemia was considered if the hematocrit level was <33%. Placental inflammation was defined as >5 mononuclear inflammatory cells observed by histological examination in 10 high-power fields using 400x magnification [46]. Low birthweight (LBW) was considered for newborns of less than 2500g, and preterm delivery for gestational ages <37 weeks.

### Statistical methods

trMPs concentration was compared between isolation methodologies using the Wilcoxon signed rank test. Student’s t test and linear regression models were used to estimate the association of HIV, malaria and other clinical and demographic covariates with log-transformed MP levels, percentages and MFIs of DC activation markers, and cytokine/chemokine concentrations in culture supernatants. Logistic regression models were used to estimate associations between adverse pregnancy outcomes (placental inflammation, preterm delivery, LBW and maternal anemia) and MP levels. Multivariate models were adjusted for maternal HIV, peripheral and placental malaria infection, parity, age and IPTp group. miRNA levels expressed as ΔΔCt were compared between infection groups by Student's t test. Statistical analysis was performed using GraphPad Prism version 6 (GraphPad Software) and Stata version 11.0 (StataCorp).

## Result

### Characteristics of the study population

The prevalence of peripheral *P*. *falciparum* infection was 15.6% (19/122). Parasites were found in 23 placental sections by histology (18.8% active infections). In total, 26 (21.3%) women presented parasites in one or both compartments by any of the techniques at the time of delivery. The characteristics of the 122 women at delivery are presented in Table 1. Fifty-six (45.9%) women received placebo and 66 (54.1%) women received SP. The subset of 122 included in this study and the 1030 women participating in the randomized trial were comparable in terms of parity, age, ITPp group, peripheral and placental malaria infection. Women with active placental malaria were younger, had lower parity and presented a higher risk of suffering peripheral malaria than non-infected. No differences were found in anemia, HIV and IPTp intervention between malaria-infected and non-infected mothers (Table 1). There were no significant differences between HIV infected and non-infected women by IPTp treatment received (P = 0.467).

**Table 1:** Demographic and clinical factors of mothers at delivery according to their placental malaria status.

		Placental malaria^a^		
		Negative (n = 99)	Active^a^ (n = 23)	P^b^
		N (%)	N (%)	
**Age (years)**		** **	** **	** **
	<20	25 (25.2)	13 (56.5)	**0.014**
	20–24	32 (32.3)	4 (17.4)	
	≥25	42 (42.4)	6 (26.1)	
**Parity**				
	Primigravidae	22 (22.2)	12 (54.5)	**0.016**
	Secundigravidae	20 (20.2)	3 (18.2)	
	Multigravidae	57 (57.6)	8 (27.3)	
**Anemia**				
	No	58 (58.6)	9 (40.9)	0.160
	Yes	40 (40.4)	13 (59.1)	
	Unknown	1 (1.01)	1 (4.54)	
**Peripheral malaria**				
	Uninfected	96 (97.0)	7 (30.4)	**<0.001**
	Infected	3 (3.03)	16 (69.6)	
**HIV infection**				
	Uninfected	50 (50.5)	11 (47.8)	0.817
	Infected	49 (49.5)	12 (52.2)	
**IPTp group**				
	Placebo	44 (44.4)	12 (52.2)	0.503
	SP	55 (55.5)	11 (47.8)	

^a^ Defined as the presence of parasites by placental histology.

^b^ Chi-square test.

Abbreviations: HIV, human immunodeficiency virus; IPTp, intermittent preventive treatment in pregnancy; SP, sulfadoxine-pyrimethamine.

### Characterization of total and trophoblast MPs

**Fig 1:**
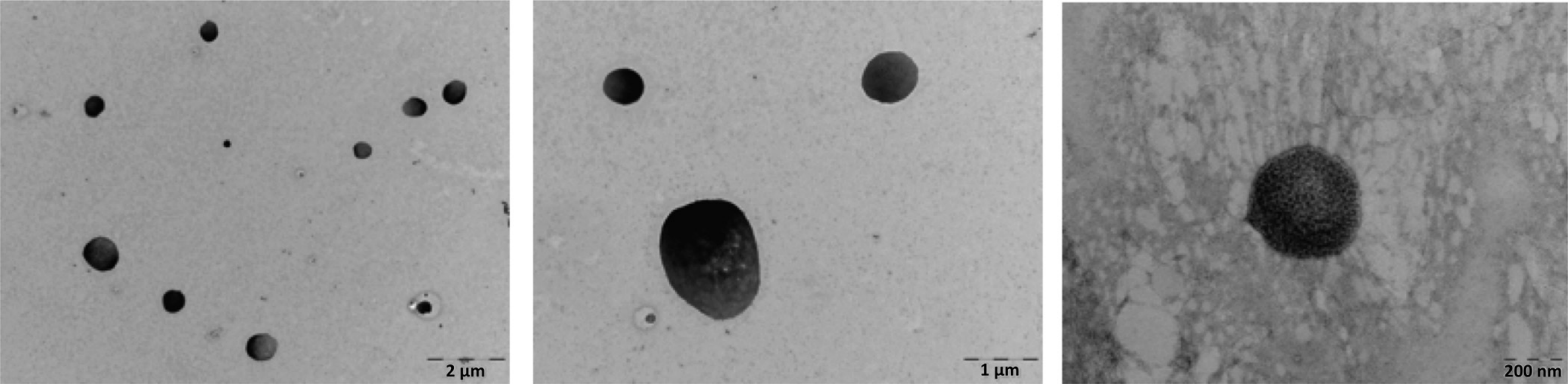
Electron microscopy imaging of negatively stained total circulating microparticles. The three images show different magnifications of the microparticles (see scale bars).

No significant difference was found in the number of trMPs per μl of plasma between centrifuged or ultracentrifuged samples (P = 0.500). However, more clearly separated Annexin V-positive populations were obtained by 10.000g centrifugation (S2 Fig), in line with previous methodologies for MP separation [4]. A high-positive Annexin V population was observed in the centrifugated samples but not when ultracentrifugation was applied, which might be explained by MP lysis when high g-force is applied (S2 Fig). By electron microscopy, it was confirmed that Annexin V-positive MPs are a population of membranous vesicles of heterogeneous size (0.2–1 μm) and morphology (Fig 1).

### Association of circulating MP levels with infection and pregnancy outcomes

**Fig 2:**
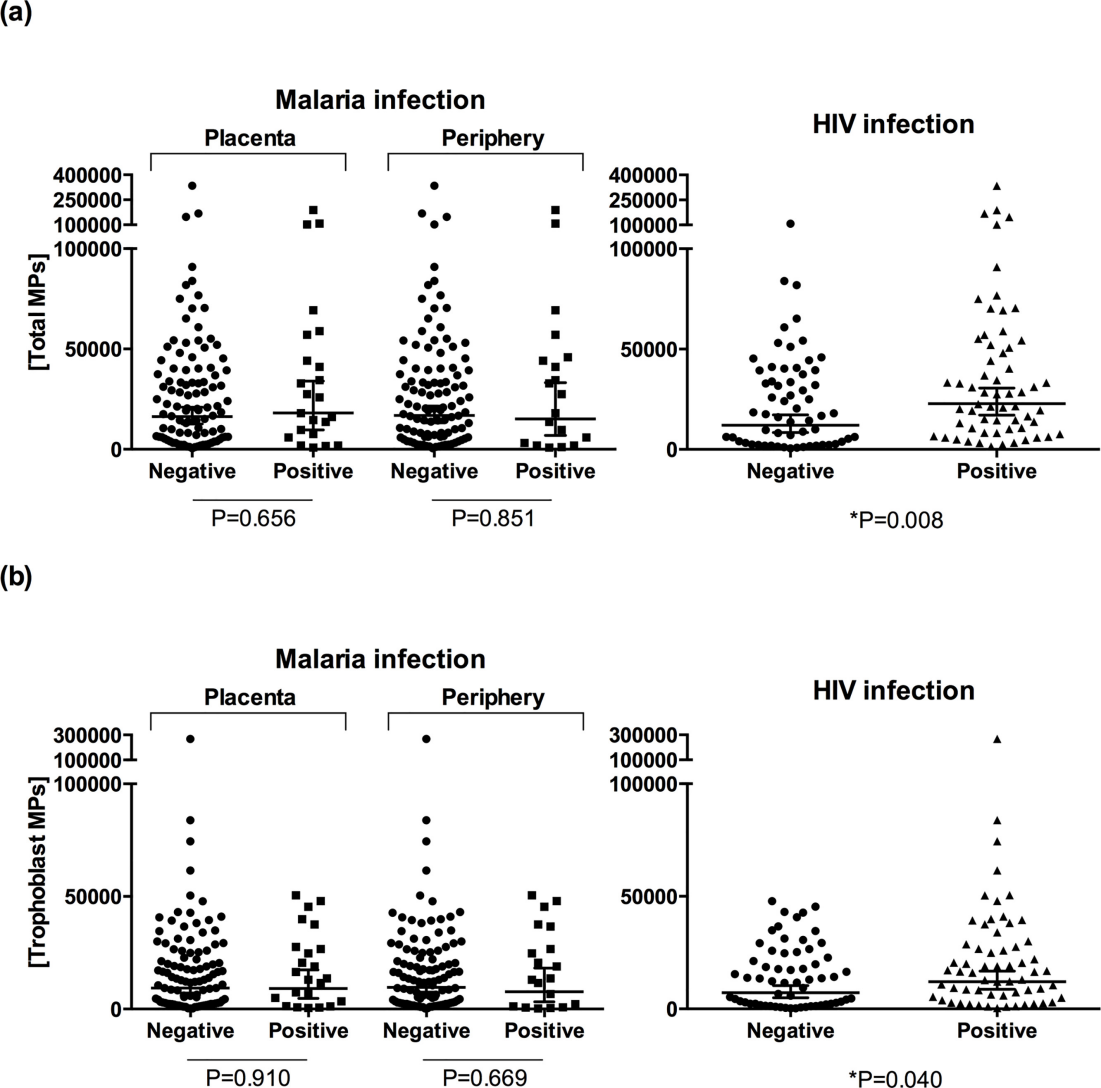
Concentrations of total (a) and trophoblast (b) MPs by maternal infectious status (n = 122). Concentrations measured by flow cytometry are expressed as number of MPs per μl of plasma. Horizontal lines represent the geometric mean and the 95% confidence interval. P-values were obtained from the univariate analysis. Significant differences maintained in the multivariate analysis are indicated by an asterisk.

Numbers of total (Fig 2A) and trMPs (Fig 2B) per μl of plasma for the 122 women were compared according to their infectious status. In a univariate analysis, concentrations of total MPs and trMPs were higher in HIV-infected than in non-infected women (P = 0.008 and P = 0.040, respectively), and the significant differences were maintained in the multivariate analysis (1.99-fold increase, 95% CI 1.24–3.21, P = 0.005; and 1.77-fold increase, 95% CI 1.06–2.96, P = 0.030, respectively).

No significant differences were found between women with and without placental malaria in the number of total MPs (P = 0.656) or trMPs (P = 0.910) per μl of plasma. Similarly, no significant differences were found in total MP (P = 0.851) or trMP (P = 0.669) concentration between women with or without peripheral malaria, neither among age (total MPs: P = 0.434 and P = 0.626 for age groups 20–24 or ≥25 compared with <20, respectively; and trMPs: P = 0.341 and P = 0.567 for age groups 20–24 or ≥25 compared with ≤20, respectively) or parity groups (total MPs: P = 0.692 and P = 0.677 for secundigravidae or multigravidae compared with primigravidae, respectively; and trMPs: P = 0.568 and P = 0.892 for secundigravidae or multigravidae compared with primigravidae, respectively).

**Fig 3:**
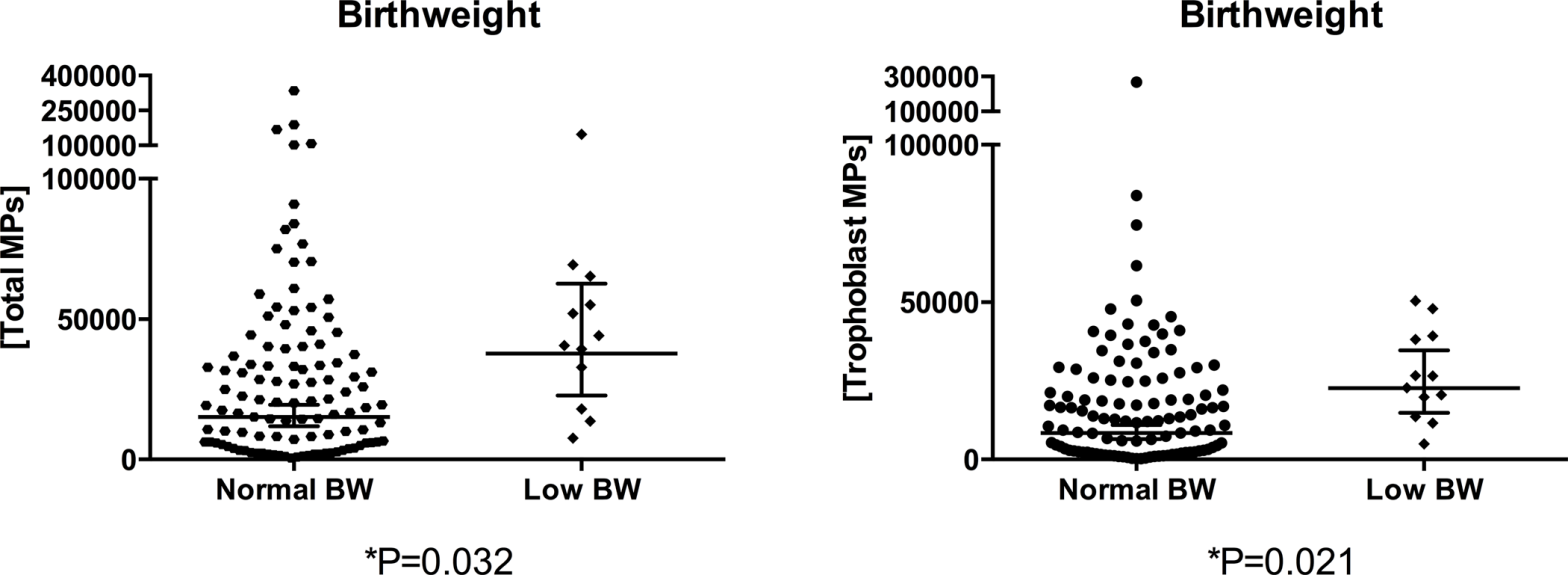
Concentrations of total and trophoblast MPs by newborn's birthweight (BW) (n = 122). Concentrations are expressed as number of MPs per μl of plasma. Horizontal lines represent the geometric mean and the 95% confidence interval. P-values were obtained from the univariate analysis. Significant differences maintained in the multivariate analysis are indicated by an asterisk.

The association between pregnancy outcomes and MP concentrations was analysed in a univariate model. Women delivering LBW babies had higher levels of total MP (P = 0.032) and trMP (P = 0.021) than mothers delivering normal birthweight newborns (Fig 3). The association was maintained in the multivariate analysis both for concentrations of total MP (odds ratio, OR = 2.38 with doubling levels of MPs; 95% CI, 1.08–5.25; P = 0.032) and trMP (OR = 2.17; 95% CI, 1.06–4.46; P = 0.034), and was therefore independent from maternal HIV or malaria infections. No associations with maternal anemia (53 women), placental inflammation (66 women) or preterm delivery (3 women) were found.

### Placental miRNA content of circulating MPs

**Fig 4:**
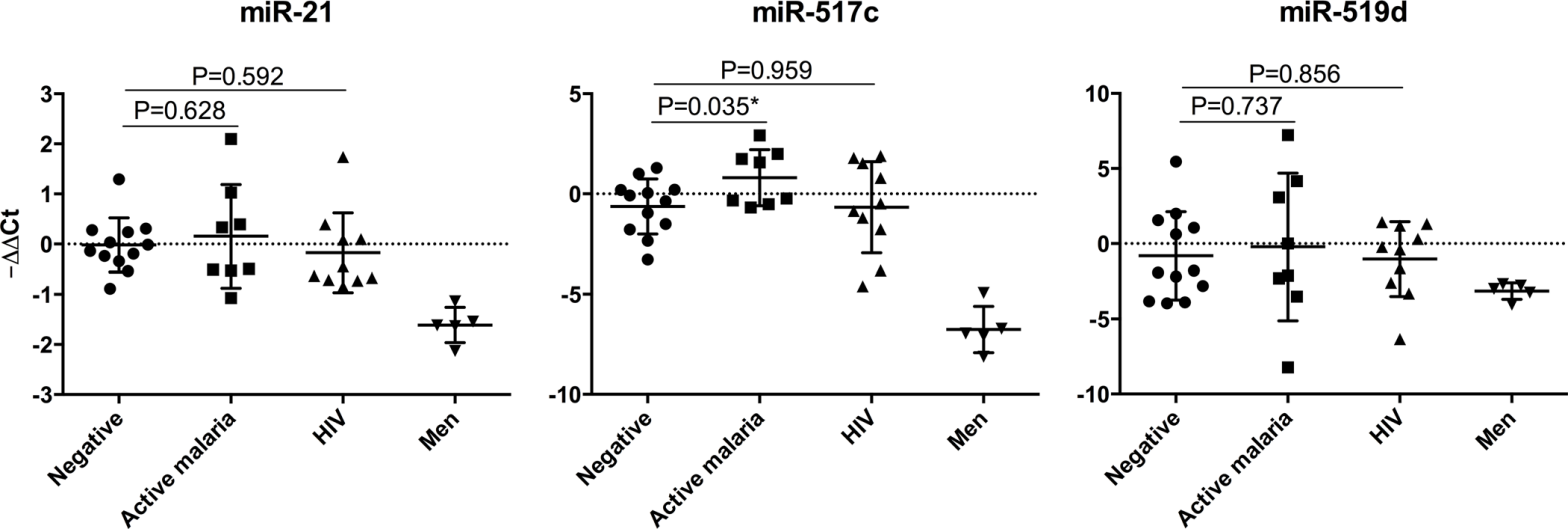
Expression of placental microRNAs in circulating microparticles by maternal infection status. MicroRNA expression levels were calculated with the delta delta Ct method. Non-infected women microparticle results were used as calibrators and miR-191 as endogenous control. Twelve women were included in the negative group (negative both for malaria and HIV), 8 in the active placental malaria and 10 in the HIV-infected group. Five men were included as negative controls for the expression of placental-related microRNAs. Means and standard deviations are represented. Statistically significant differences from Student's t test are marked with an asterisk. miR-493 was only expressed in 4/30 (13.3%) of the women samples, and therefore not included in the analysis by maternal infection status. There were no significant differences in miRNA expression between women with or without placental inflammation (P = 0.581 for miR-21, P = 0.695 for miR-517c and P = 0.989 for miR-519d). Only one woman delivering a LBW baby was included in the miRNA study, and therefore a comparison between LBW and normal birthweight delivering mothers was not possible. No statistically significant correlations were found between birthweight as a continuous variable and miRNA expression.

The 30 women included in the miRNA sub-study were comparable with the whole study cohort in terms of age, parity, malaria and HIV infections, IPTp, anemia and low birthweight babies, with the exception of placental inflammation that was more prevalent in the whole cohort (S1 Table). No significant hemolysis was observed in any of the 30 plasma samples used for miRNA quantification [Median A_414_ = 0.081, interquartile range, IQR 0.067–0.105; median haemoglobin concentration (g/L) = 0.153, IQR 0.052–0.318]. miR-191 and miR-21 were detected in all MP samples isolated from pregnant women. miR-517c was detected in 87.5% (7/8) of MPs from women with active placental malaria, compared with 41.7% (5/12) of non-infected (P = 0.040) and 50% (5/10) of HIV-infected women (P = 0.093). miR-519d was expressed in MPs from 66.7% of non-infected, 75% of active malaria and 70% of HIV infections (P = 0.924). miR-493 expression was only detected in 8% of non-infected, 25% of mothers with placental malaria and 10% of women with HIV (P = 0.522) and not further included in the analysis. Raw Cts by infection status are shown in S3 Fig. When ΔΔCts for each miRNA normalized with respect to miR-191 were compared among infected and non-infected women (Fig 4), miR-517c expression was found to be higher in MPs from mothers with active placental malaria than in the non-infected group (P = 0.035).

### Functional effect of trophoblast MPs on dendritic cells

**Fig 5:**
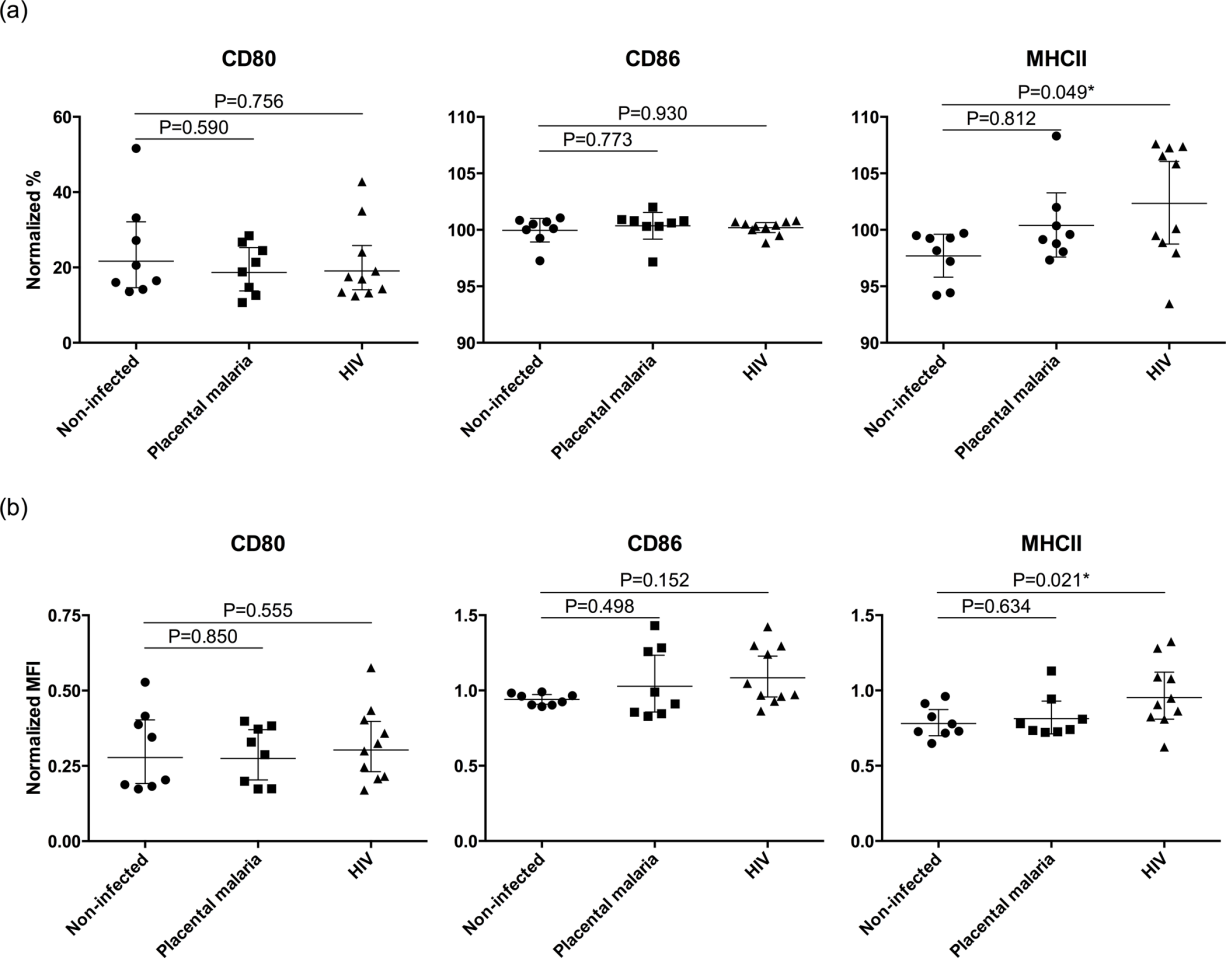
Phenotype of dendritic cells after incubation with circulating microparticles (n = 26). The normalized percentage of dendritic cells expressing an activation marker (a) or the normalized mean fluorescence intensity (MFI) (b) are represented as circles (negative both for malaria and HIV), squares (active malaria-infected) or triangles (HIV-infected). Percentages and MFIs were normalized with respect to the positive control incubated with lipopolysaccharide. Geometric means and 95% confidence intervals are represented. P-values correspond to the univariate analysis. Statistically significant differences maintained after adjustment in the multivariate model are marked with an asterisk.

After incubation for 24h with MPs isolated from 26 plasmas, DCs expressed the activation markers CD80, CD86 and MHCII (Fig 5), although there were no significant differences in the expression of CD80 and CD86 between DCs incubated with MP_NI_, MP_malaria_ or MP_HIV_. In the univariate analysis, a higher percentage of DCs incubated with MP_HIV_ expressed MHCII (P = 0.049) and levels of MHCII expression were higher (P = 0.021), when compared with DCs stimulated with MP_NI_. After adjustment, the percentage of DCs expressing MHCII remained significantly augmented when incubating with MP_HIV_ (1.05-fold increase; 95% CI, 1.00–1.10; P = 0.041) compared to MP_NI_, and the MHCII MFI was increased but with borderline significance (1.22-fold increase; 95% CI, 0.988–1.51; P = 0.063).

**Fig 6:**
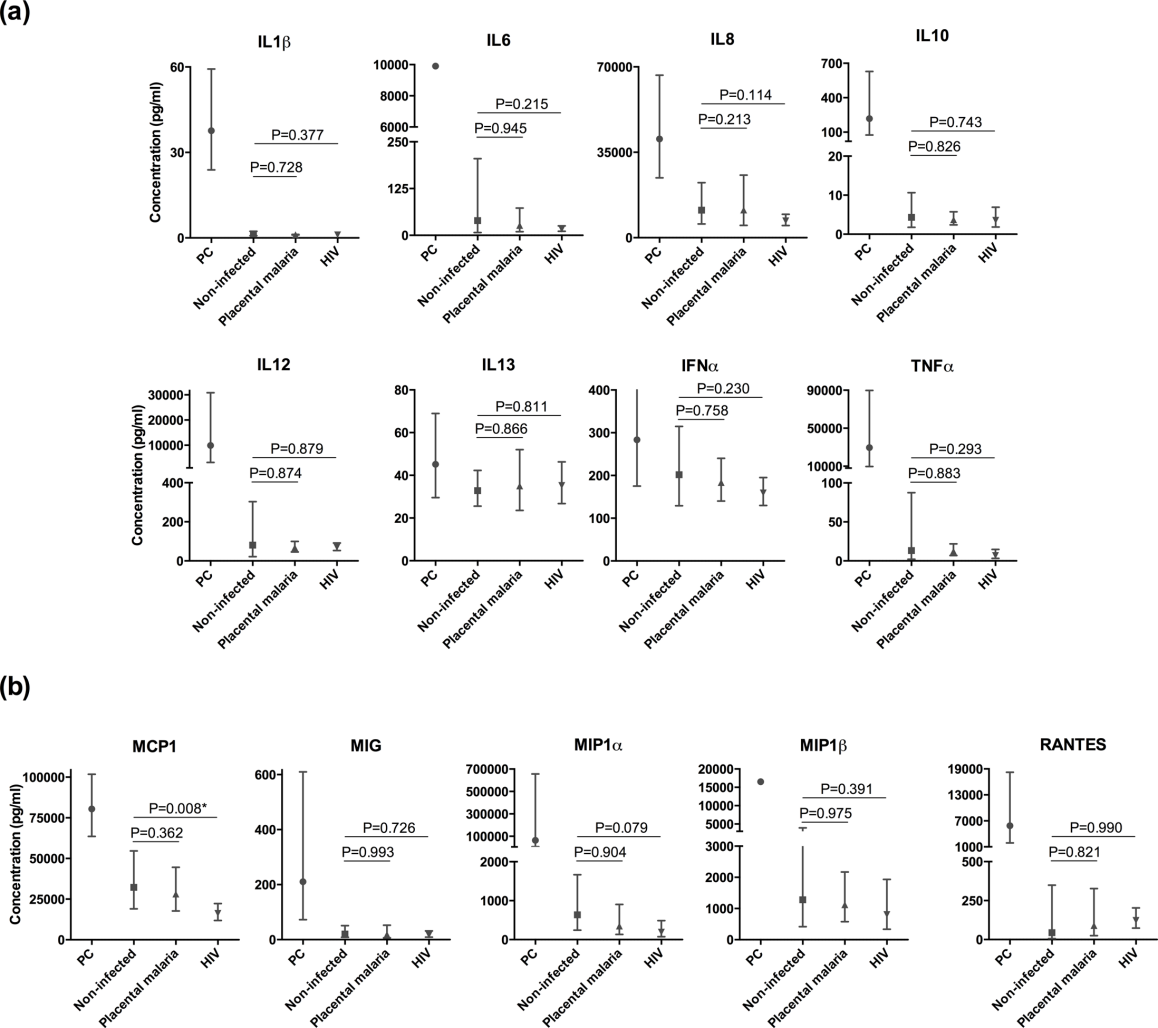
Cytokine (a) and chemokine (b) concentrations in dendritic cell culture supernatants after incubation with MPs. Twenty-six women were included. Concentrations are expressed in pg/ml. PC represents the positive control condition with only lipopolysaccharide. Geometric means and 95% confidence intervals are represented. P-values correspond to the univariate analysis. Statistically significant differences maintained after adjustment in the multivariate model are marked with an asterisk.

Several cytokines and chemokines were detected in DC culture supernatants after incubation with MPs, although, in general, there were not significant differences among MP_NI_, MP_malaria_ or MP_HIV_ alone (Fig 6) or after simultaneous incubation of MPs with LPS (S4 Fig). Only MP_HIV_ induced lower production of *Monocyte Chemoattractant Protein-1* (MCP1) in DC supernatants (0.539-fold decrease; 95% CI, 0.346–0.838; P = 0.008) than MP_NI_ in the univariate analysis, and the difference was maintained after adjustment (0.426-fold decrease; 95% CI, 0.219–0.827; P = 0.015). There was a trend towards a higher expression of IL13 when incubating DCs with MPs and LPS than with LPS alone, although not statistically significant.

## Discussion

This is the first study that, to our knowledge, analyses the relationship of malaria and HIV infection in pregnancy with alterations in circulating MPs and their miRNA content. Total and trMPs were increased in women infected with HIV, which *in vitro* led to increased expression of MHCII and reduced MCP1 in DCs, and in mothers delivering LBW babies, compared to MPs from non-infected or those delivering normal birthweight newborns, respectively. Although malaria in pregnant women was not associated with changes in circulating MPs, the expression of pregnancy-related microRNAs (miR-517c) in MPs was higher in active placental malaria than in uninfected mothers. Overall, it is plausible to speculate a role for trMPs and placental miRNAs in the pathophysiology of malaria and HIV infections during pregnancy.

The augmented levels of total MPs in HIV-positive women could be explained by platelet activation and increase of procoagulant MPs associated with HIV infection [47,48]. HIV-related changes in placental membranes [31] might lead to increased concentration of trMPs and contribute to the augmented risk of LBW reported among HIV-infected pregnant women [49]. In line with previous reports showing increased levels of MPs, including placental MPs, in pre-eclampsia [23,50], IUGR [51] and recurrent miscarriage [52], as well as in endothelial activation and induction of a procoagulant state [53], the present results support a crucial role for MPs in placenta dysfunction and vascular pregnancy complications [52]. Similar mechanisms may contribute to MP-dependent LBW in HIV-infected pregnant women. As placental MPs gradually increase from approximately 10 weeks of gestation [20], their measurement might be useful for the identification of pregnancies with LBW risk at early stages of gestation. The increase of placental MPs associated with inflammation in pathologies such as pre-eclampsia [23] was not observed in women with placental inflammation from our cohort. The effect of placental inflammation on MP release should be addressed in future studies analysing cytokines and other inflammatory mediators in the mothers.

In contrast to HIV, placental malaria was not associated with changes in MP concentrations, suggesting placental alterations and molecular mechanisms different than those observed for MPs in HIV infection. However, malaria may be associated with changes in vesicle miRNA content. miR-517c from the cluster C19MC, previously shown to have a role as immunomodulator in pregnancy and tumorigenesis [54], was found overexpressed in mothers with placental malaria compared with non-infected. Our study suggests an interesting diagnostic potential for pregnancy-specific miRNAs from C19MC [41], in line with reports showing an augment of miR-517c expression with increasing placental weight [55] and an overexpression in pre-eclampsia [26] and recurrent spontaneous abortion [56], that should be addressed in future studies.

MPs from HIV-infected mothers induced a higher expression of MHCII in DCs than MPs from uninfected women, suggesting a modulatory effect in DC function. Moreover, the increased MHCII expression in DCs after incubation with MPs from HIV-infected women without upregulation of co-stimulatory molecules, as well as the trend towards a higher expression of IL13 in DCs after incubation with MPs compared with LPS alone, is in line with the previously proposed tolerogenic role of MPs in pregnancy [21,22]. Although the changes induced by MPs in our DC system are modest and their biological relevance needs confirmation, they are indicative of an *in vitro* effect over immune cells even when only 20 μl of plasma are used as starting material. Higher numbers of MPs are expected to have increased stimulatory capacity and, therefore, a more pronounced effect over DCs that should be determined in future studies.

Our study presents several limitations. First, EVs are a highly heterogeneous population, making difficult to completely discard some contaminations with exosomes, apoptotic bodies or other plasma components during MP preparation. Since the miRNA analysis was performed in a small number of women and miRNAs, further studies are needed including more samples and profiling methodologies. Other potential maternal infections, endogenous factors or environmental exposures that could affect MP release or miRNA expression should be taken into account in future works. Since we chose to incubate DCs with MPs obtained from the same volume of plasma from all the participants to reproduce physiological conditions where circulating MP concentrations are variable, the effect of normalized amounts of MPs was not addressed. Moreover, we did not correct for multiple comparisons when assessing the effect of MPs on cytokine/chemokine production by DCs, although in general there were no significant differences after incubation with MP_NI_, MP_malaria_ or MP_HIV_, with the exception of the decrease in MCP1 with MP_HIV_, that was consistently maintained in the multivariate analysis. Finally, the cross-sectional nature of this work describes potential associations but causal relationships cannot be inferred.

In summary, this study shows that plasma levels of total and trophoblast-derived MPs, which are increased in HIV-infected pregnant women and in those delivering LBW newborns, can alter the *in vitro* function of immune regulatory cells such as DCs. In contrast, active placental malaria is associated with changes in miRNA of placental origin contained in circulating MPs. Both MPs and miRNAs might affect the immunoregulatory balance during pregnancy, although further studies to unravel the mechanisms are required. Importantly, the results of this study suggest that placental miRNAs might present a utility as biomarkers of placental malaria infection during pregnancy that should be further explored and validated. The characterization of different vesicle populations and their content may increase our understanding of immunopathological processes in malaria and HIV and guide the discovery of new biomarkers for malaria in pregnancy, an infection that frequently remains undetected but still has deleterious consequences [57].

## Supporting Information

S1 Fig Gating strategy for the quantification of total and trophoblast microparticles. A. Regions corresponding to calibration beads of 0.2, 0.5 and 1 μm of diameter are shown. B. All the events in the sample indicating the regions for the calibration beads and the background (BG). C. Events with forward scatter patterns (FSC-A) between those obtained with fluorescent calibration microspheres of diameters 0.2 and 1 μm (left) positively stained for Annexin V (right) were considered total circulating microparticles (in this particular example, 89.6% of the total events after background exclusion). D. Unstained control sample showing the gate for the annexin V and PSG1 positive population. E. Events that were positive both for Annexin V and PSG1 staining were considered trophoblast microparticles (30.4% of the population of particles 0.2–1 μm in the example).
(TIFF)

S2 Fig Comparison of circulating microparticles isolation methodologies. Example of gating and percentages of the Annexin V-positive population after isolation of circulating microparticles from plasma by centrifugation (CENT) at 10.000g (A, unstained control; B, annexin V stained sample) or ultracentrifugation (ULTRA) at 100.000g (C, unstained control; D, annexin V stained sample).
(TIFF)

S3 Fig Raw cycle thresholds (Cts) of placental microRNAs determined by qPCR in circulating microparticles samples. Non-infected women are represented by circles, women with active placental malaria by squares and HIV-positive mothers by triangles. Means and standard deviations are represented. A value of Ct = 41 was arbitrarily assigned to samples where the expression of a microRNA was not detected after 40 qPCR cycles.
(TIFF)

S4 Fig Cytokine (a) and chemokine (b) concentrations in dendritic cell culture supernatants after incubation with microparticles plus lipopolysaccharide. Twenty-six women were included. Concentrations are expressed in pg/ml. PC represents the positive control condition with only lipopolysaccharide. Geometric means and 95% confidence intervals are represented. Statistical analysis did not reveal statistical differences among the groups and therefore P-values are not indicated (P≥0.005).
(TIFF)

S1 Table Comparison of demographic and clinical factors between mothers included in the microRNA subset and the whole study cohort. (DOC)
