# Supplementary figures and images for: Placental Microparticles and MicroRNAs in Pregnant Women with Plasmodium falciparum or HIV Infection
Source: PLoS One. 2016 Jan 12;11(1):e0146361. doi: 10.1371/journal.pone.0146361 (PMC4710532; doi:10.1371/journal.pone.0146361)

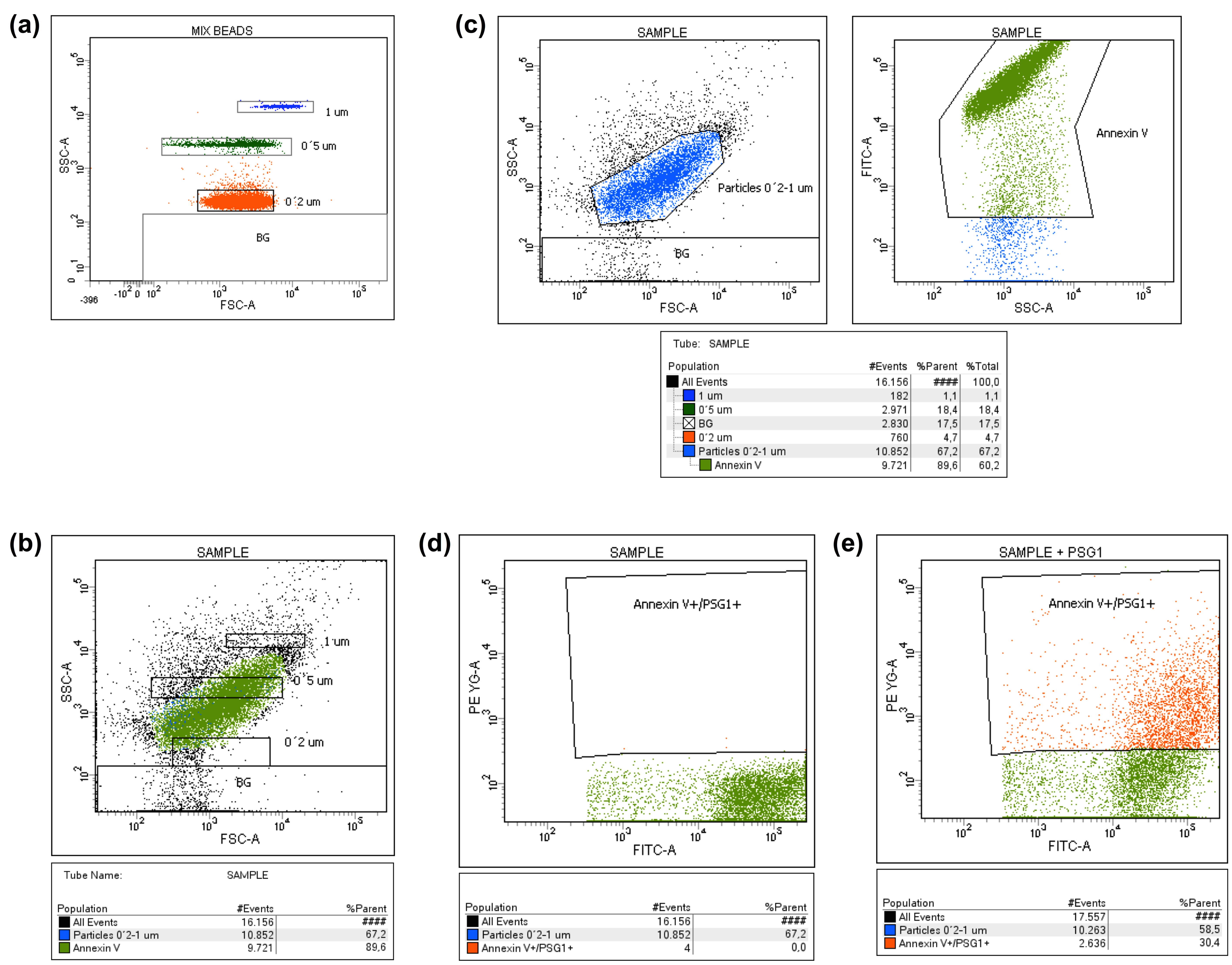

Supplement: S1 Fig — A. Regions corresponding to calibration beads of 0.2, 0.5 and 1 μm of diameter are shown. B. All the events in the sample indicating the regions for the calibration beads and the background (BG). C. Events with forward scatter patterns (FSC-A) between those obtained with fluorescent calibration microspheres of diameters 0.2 and 1 μm (left) positively stained for Annexin V (right) were considered total circulating microparticles (in this particular example, 89.6% of the total events after background exclusion). D. Unstained control sample showing the gate for the annexin V and PSG1 positive population. E. Events that were positive both for Annexin V and PSG1 staining were considered trophoblast microparticles (30.4% of the population of particles 0.2–1 μm in the example). (TIFF) [file pone.0146361.s001.tiff]

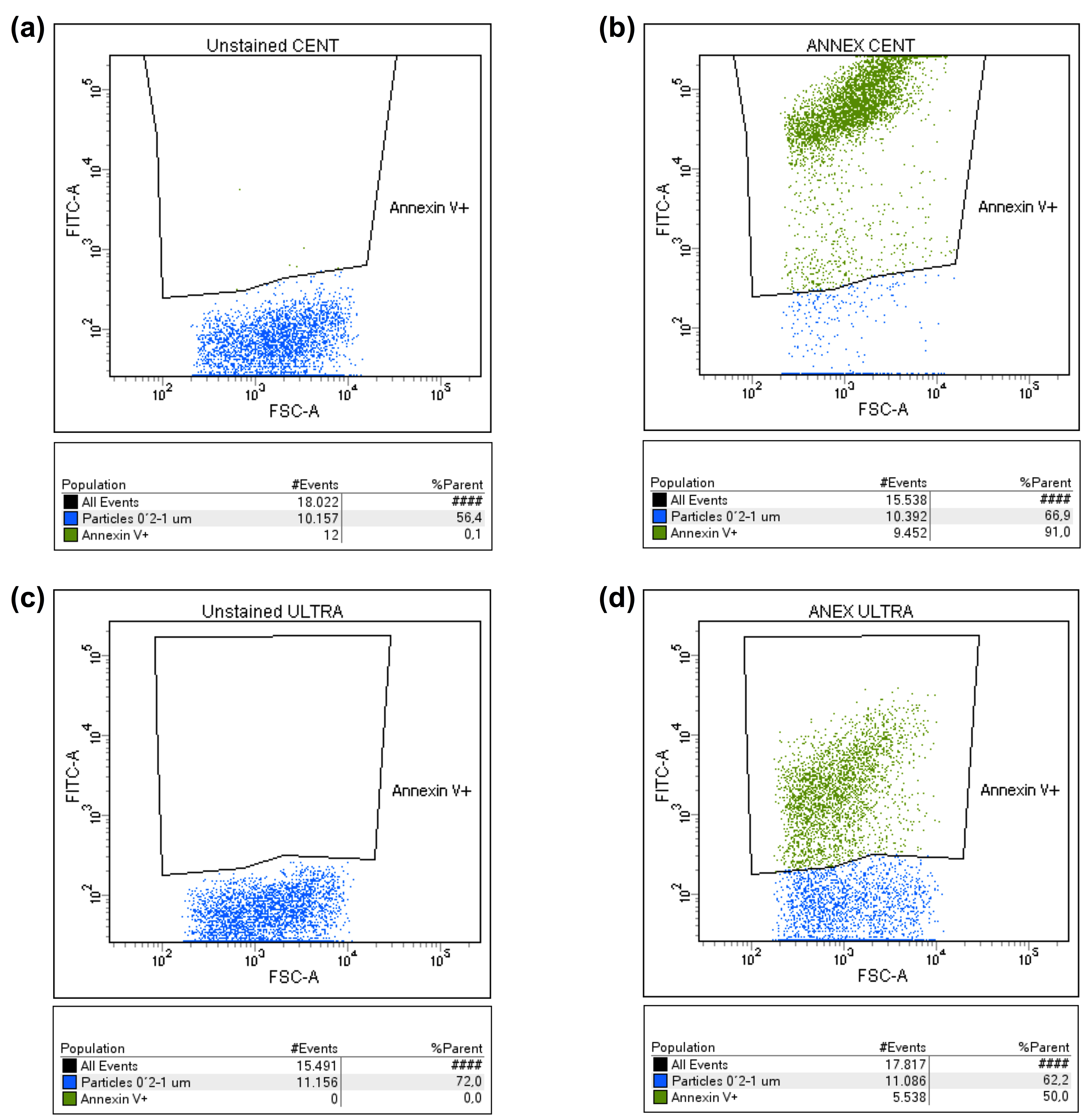

Supplement: S2 Fig — Example of gating and percentages of the Annexin V-positive population after isolation of circulating microparticles from plasma by centrifugation (CENT) at 10.000g (A, unstained control; B, annexin V stained sample) or ultracentrifugation (ULTRA) at 100.000g (C, unstained control; D, annexin V stained sample). (TIFF) [file pone.0146361.s002.tiff]

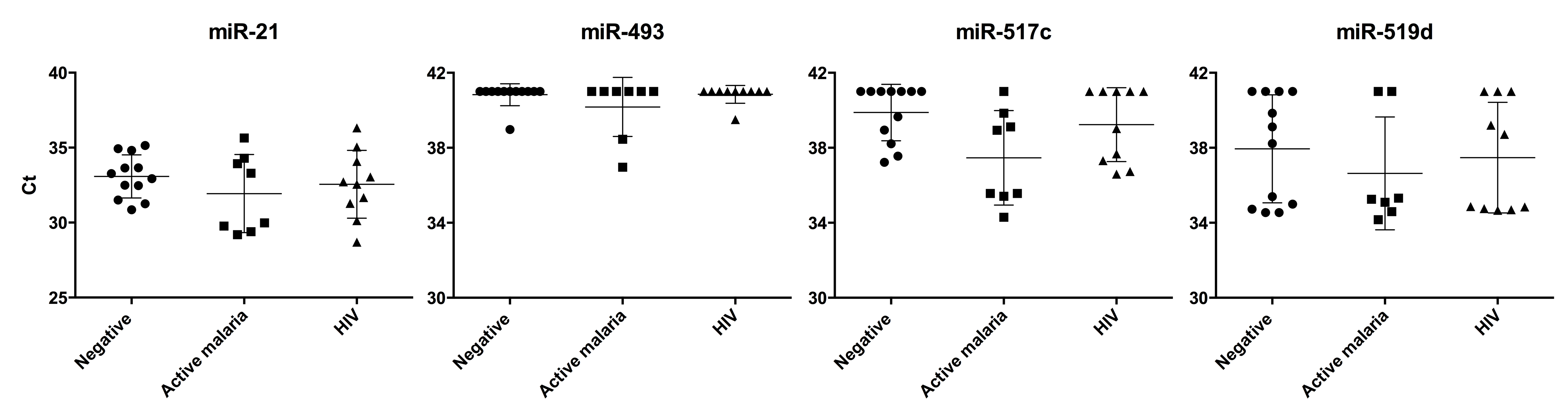

Supplement: S3 Fig — Non-infected women are represented by circles, women with active placental malaria by squares and HIV-positive mothers by triangles. Means and standard deviations are represented. A value of Ct = 41 was arbitrarily assigned to samples where the expression of a microRNA was not detected after 40 qPCR cycles. (TIFF) [file pone.0146361.s003.tiff]

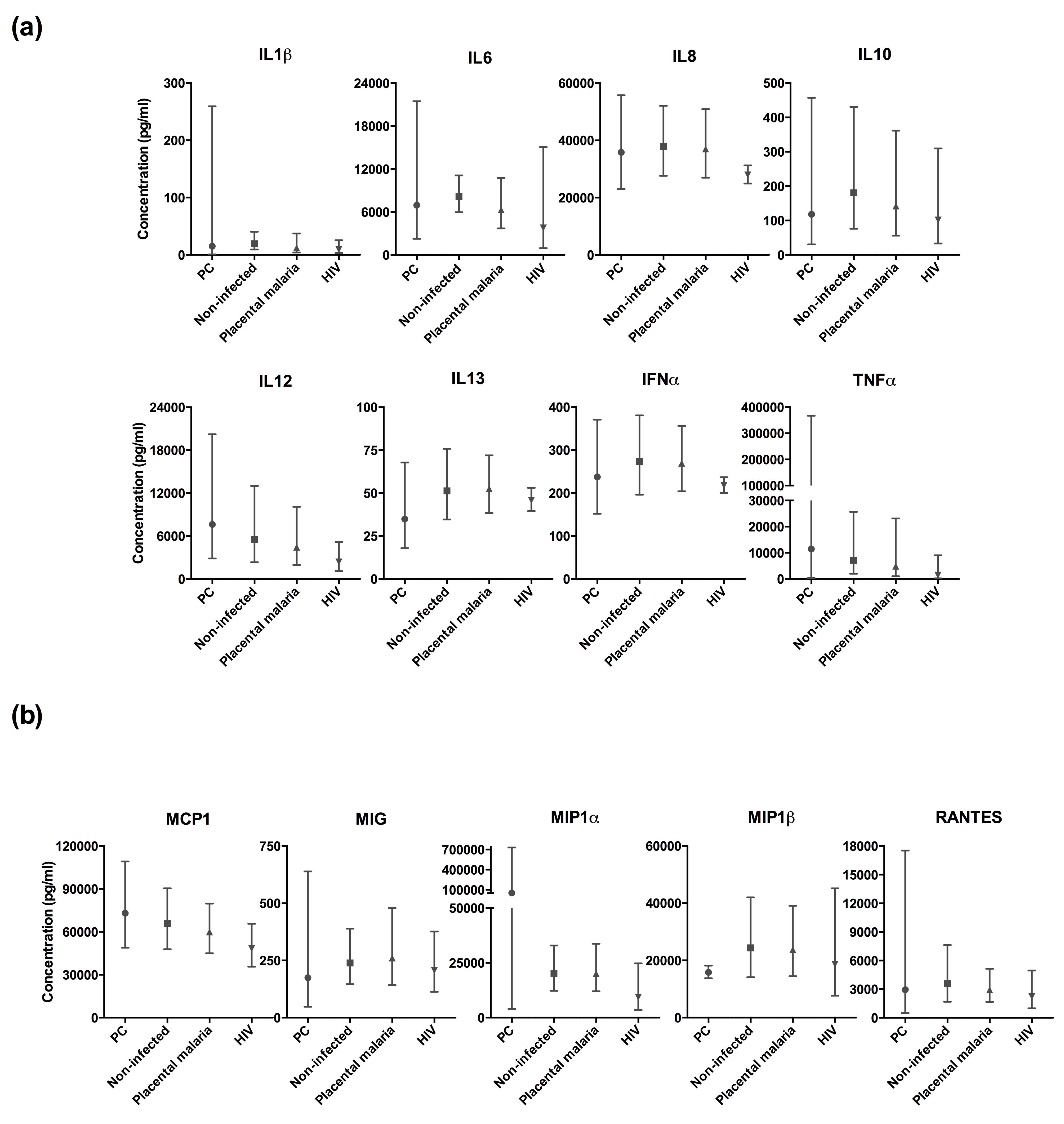

Supplement: S4 Fig — Twenty-six women were included. Concentrations are expressed in pg/ml. PC represents the positive control condition with only lipopolysaccharide. Geometric means and 95% confidence intervals are represented. Statistical analysis did not reveal statistical differences among the groups and therefore P-values are not indicated (P≥0.005). (TIFF) [file pone.0146361.s004.tiff]
