# Supplementary material for: Placental Microparticles and MicroRNAs in Pregnant Women with Plasmodium falciparum or HIV Infection
Source: PLoS One. 2016 Jan 12;11(1):e0146361. doi: 10.1371/journal.pone.0146361 (PMC4710532; doi:10.1371/journal.pone.0146361)
Supplement: S1 Table — (DOC) [file pone.0146361.s005.doc]

**S1 Table. Comparison of demographic and clinical factors between mothers** included in the microRNA subset and the whole study cohort.

|  |  | **Nº women from miRNA subset (n=30)** | **Nº women from whole cohort (n=122)** | **P^a^** |
| --- | --- | --- | --- | --- |
| **Age (years)** |  |  |  |  |
|  | <20 | 8 | 28 | 0.970 |
|  | 20- 24 | 9 | 36 |
|  | ≥25 | 13 | 48 |
| **Parity** | |  |  |  |
|  | Primigravidae | 8 | 34 | 0.858 |
|  | Secundigravidae | 7 | 23 |
|  | Multigravidae | 15 | 65 |
| **Placental malaria** | |  |  |  |
|  | Uninfected | 20 | 99 | 0.085 |
|  | Infected | 10 | 23 |
| **Peripheral malaria** | |  |  |  |
|  | Uninfected | 22 | 103 | 0.154 |
|  | Infected | 8 | 19 |
| **HIV infection** | |  |  |  |
|  | Uninfected | 20 | 61 | 0.101 |
|  | Infected | 10 | 61 |
| **IPTp group** | |  |  |  |
|  | Placebo | 17 | 54 | 0.252 |
|  | SP | 13 | 66 |
| **Anemia** |  |  |  |  |
|  | No | 19 | 67 | 0.536 |
|  | Yes | 10 | 53 |
|  | Unknown | 1 | 2 |
| **Low birthweight** |  |  |  |  |
|  | No | 29 | 110 | 0.254 |
|  | Yes | 1 | 12 |
| **Placental inflammation** |  |  |  |  |
|  | No | 24 | 54 | **<0.001** |
|  | Yes | 6 | 68 |

^a^ Chi-square test.

Abbreviations: HIV, human immunodeficiency virus; IPTp, intermittent preventive treatment in pregnancy; SP, sulfadoxine-pyrimethamine.
